# Gastric Volvulus: Diagnosis and Successful Endoscopic De-rotation Towards Conservative Management in a Patient With Multiple Comorbidities

**DOI:** 10.7759/cureus.26862

**Published:** 2022-07-14

**Authors:** Mansoor Zafar, Joe Parvin, Alexandra Mcwhirter, Linda Loterh, Indu Koshi, Tatyana Viner, Gareth Watts, Kofi Ofuafor

**Affiliations:** 1 Gastroenterology, Hepatobiliary, Hepatology, General Internal Medicine, Royal Sussex County Hospital, University Hospitals Sussex NHS Foundation Trust, Brighton, GBR; 2 Gastroenterology, General Internal Medicine, Royal Sussex County Hospital, University Hospitals Sussex NHS Foundation Trust, Brighton, GBR; 3 General Internal Medicine, Royal Sussex County Hospital, University Hospitals Sussex NHS Foundation Trust, Brighton, GBR; 4 Acute Medicine, Royal Sussex County Hospital, University Hospitals Sussex NHS Foundation Trust, Brighton, GBR; 5 Geriatric Medicine, Princess Royal Hospital, University Hospitals Sussex NHS Foundation Trust, Haywards Heath, GBR; 6 Acute Medicine, General Internal Medicine, Royal Sussex County Hospital, University Hospitals Sussex NHS Foundation Trust, Brighton, GBR

**Keywords:** ogd - oesophago gastroduodenoscopy, increased comorbidities, de-rotation, para-oesophageal hiatus hernia, gastric volvulus

## Abstract

Gastric volvulus is a condition that is not frequently seen in clinical practice and can present with a myriad of symptoms, meaning it can be challenging to diagnose. We present an 82-year-old female attending the emergency department with epigastric pain and coffee ground vomiting on a background of rectosigmoid cancer and a large, complex hiatus hernia. On investigation there was no drop in haemoglobin. However, the chest X-ray showed air-fluid levels and raised the suspicion of gastric volvulus, particularly given her past medical history. The timely organisation of a computed tomogram (CT) scan allowed for prompt decision-making with involvement of surgical colleagues. The patient proceeded to successful conservative management with upper gastroduodenal endoscopy and a de-rotation technique. This case highlights the importance of considering gastric volvulus as a differential diagnosis in those presenting with epigastric pain and vomiting particularly in patients over 50 with a history of large hiatus hernia. This allows for prompt diagnosis and management and avoidance of major complications like gastric mucosal ischaemia.

## Introduction

Gastric volvulus is a relatively rare clinical entity. It peaks in the fifth decade, and usually, presents in the acute setting with upper abdominal epigastric pain, retching and failure to pass the nasogastric tube. This is described in the literature as Borchardt’s triad [1]. It is widely known as a demanding condition to diagnose unless the clinician has a clear understanding of and an ability to interpret the symptoms [1]. There are occasions where it does not present with failure to pass the nasogastric tube and, as seen in this case, the retching and nausea may lead to coffee ground vomiting. A background of hiatus hernia is a useful clue [1] and the incidence has been reported to peak after the fifth decade [2]. Gastric volvulus is defined as the rotation of the stomach up to 180° along its transverse or longitudinal axis and can be associated with grave outcomes if not diagnosed and managed in an appropriate time frame [3].

## Case presentation

**Figure 1:**
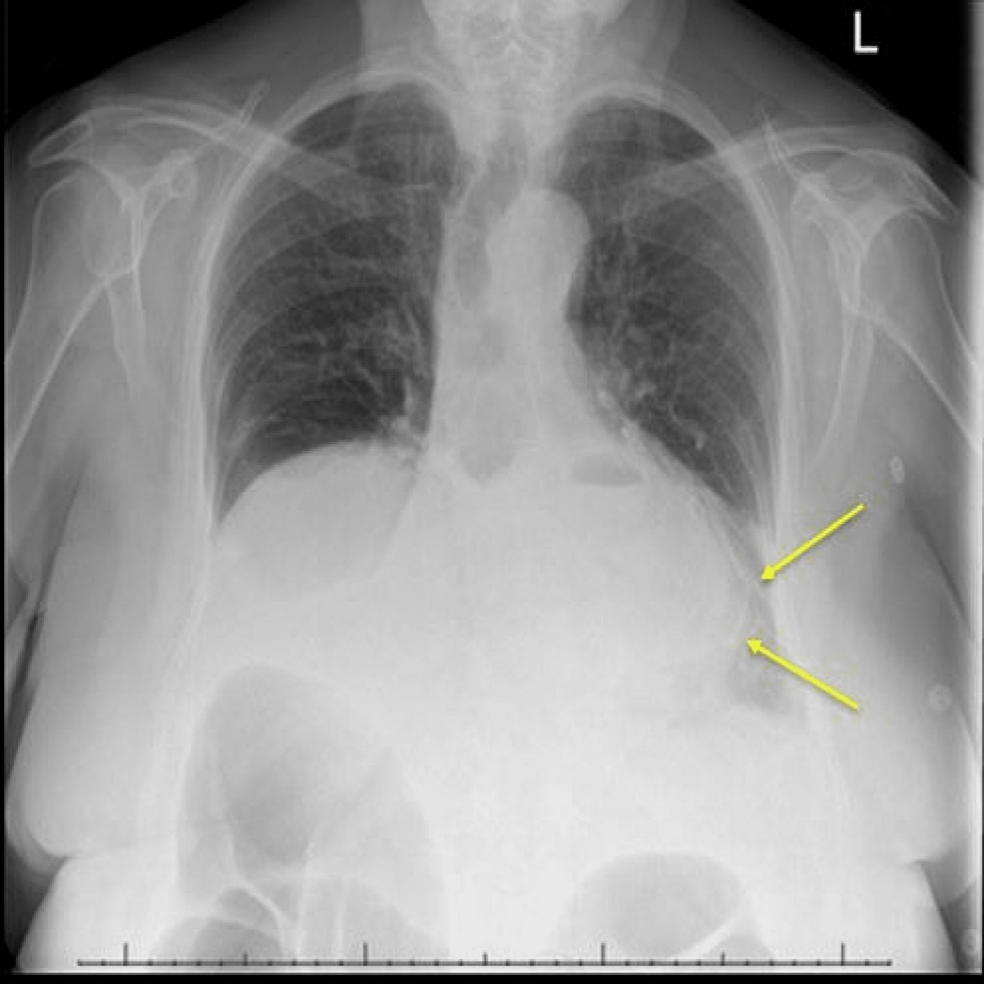
Chest x-ray with air fluid levels (yellow arrows).

An 82-year-old female with a background history of rectosigmoid cancer (successful ileostomy followed by ileoanal anastomosis), breast cancer with solitary brain metastasis to brain under oncological treatment and remote history of complex hiatus hernia of 10 centimetres (previously managed conservatively at patient’s discretion) presented to the emergency department with a three-day history of progressively worsening upper abdominal pain and nausea. During assessment, she was noticed to have retching and had an episode of coffee ground vomiting of around 200 millilitres. Her initial physical observations including blood pressure, heart rate, oxygen saturation and temperature were all within normal limits. Wide bore cannulas were urgently inserted and intravenous ondansetron 4 milligrams (antiemetic) along with intravenous omeprazole 40 milligrams were administered alongside the intravenous fluid resuscitation with 0.9% saline. Her arterial blood gas (ABG) analysis revealed no drop in her baseline haemoglobin count (133 gram/litre), which was confirmed with urgent laboratory blood test results. On physical examination she was found to have tenderness along the epigastric area with no guarding and normal bowel sounds. A nasogastric (Ryle’s) tube was inserted with draining of coffee ground contents. The patient had occasional episodes of a weak cough; therefore, an urgent request was made for a chest x-ray to investigate for evidence of aspiration (Figure 1).

**Figure 2:**
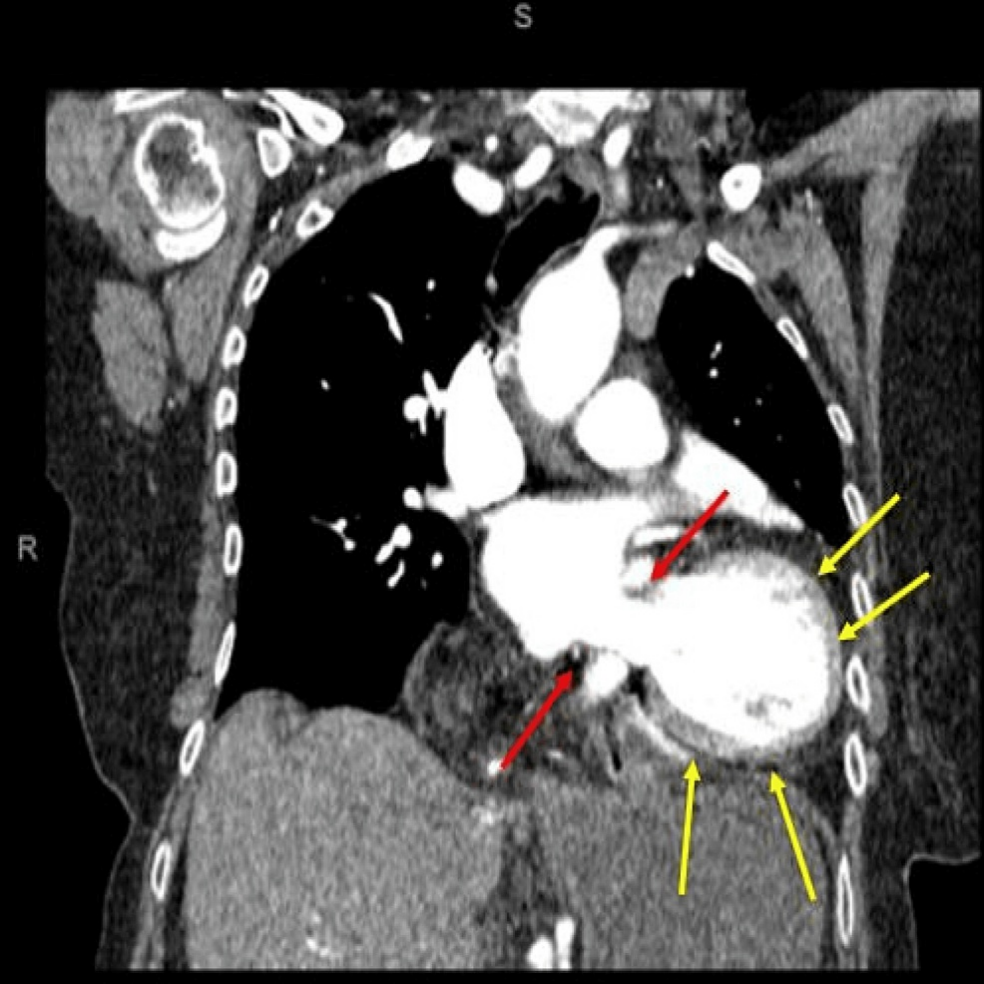
Computed tomogram (CT) chest-abdomen. Notice the gastric volvulus which appears to be associated with rolling paraesophageal type of hiatus hernia (red arrows) and fluid around the gastric component of the intra-abdominal component of the stomach (yellow arrows).

However, the chest x-ray raised suspicion for gastric volvulus. She was made nil by mouth and intravenous amoxicillin 1 gram and metronidazole 500 mg were administered to cover for aspiration pneumonia. An urgent computed tomogram of the chest and abdomen (CT chest and abdomen) was requested which also queried gastric volvulus (Figure 2).

**Figure 3:**
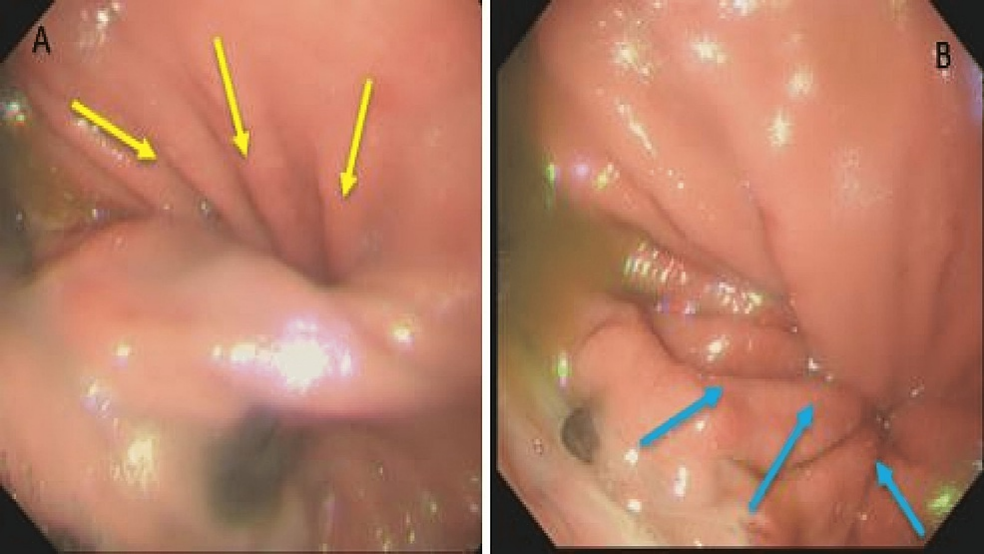
Gastric volvulus with rolling (para-oesophageal hernia). A) Clockwise rotation, making further antral progression of endoscope near impossible (yellow arrows). B) Anticlockwise torquing of advancing endoscope initiated while clockwise volvulus is seen (blue arrows).

The case was initially discussed with the surgical team, however considering the patient’s comorbidities, a decision was made for conservative management in the first instance using upper gastroduodenal endoscopy. Gastric volvulus with clockwise rotation was confirmed (Figure 3A). Anticlockwise torquing was initiated via an advancing endoscope attempting to go past the volvulus segment towards the gastric antrum (Figure 3B).

**Figure 4:**
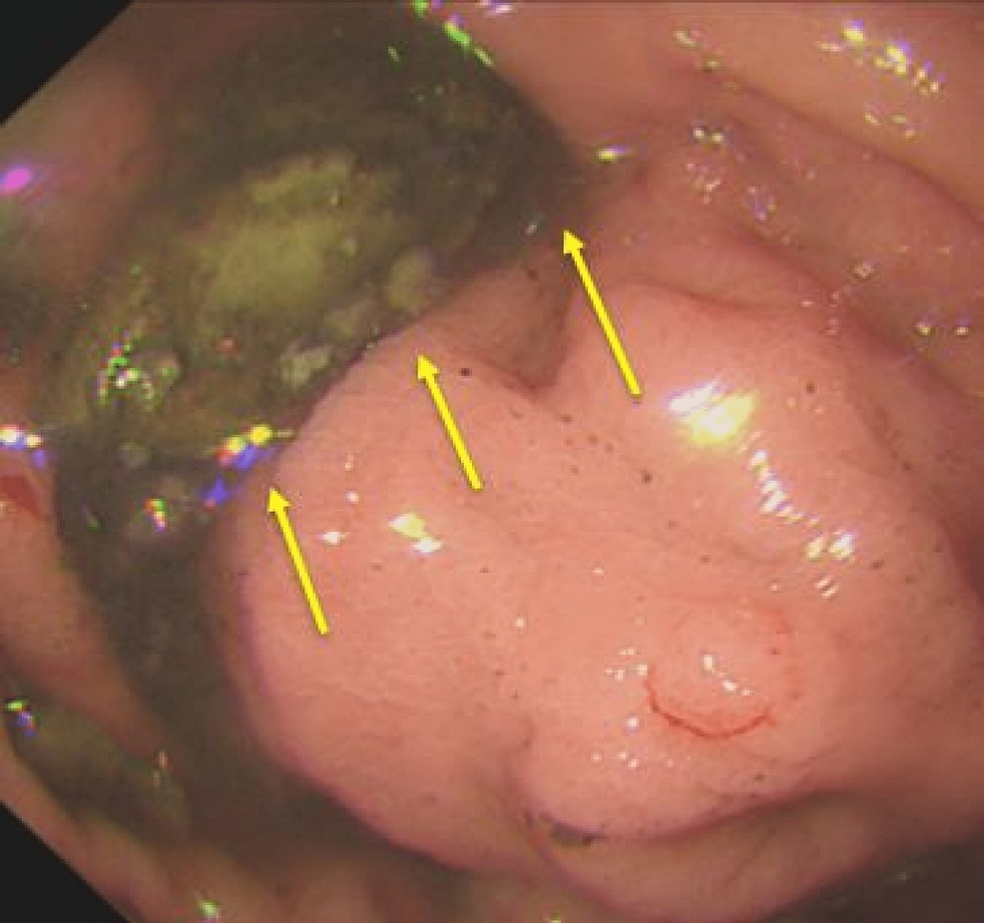
Suctioning of residual food debris (yellow arrows) while anticlockwise torquing via advancing endoscope continues.

While the endoscope was advancing, attempting to de-rotate with anticlockwise torquing, simultaneous suctioning of residual food debris continued (Figure 4).

**Figure 5:**
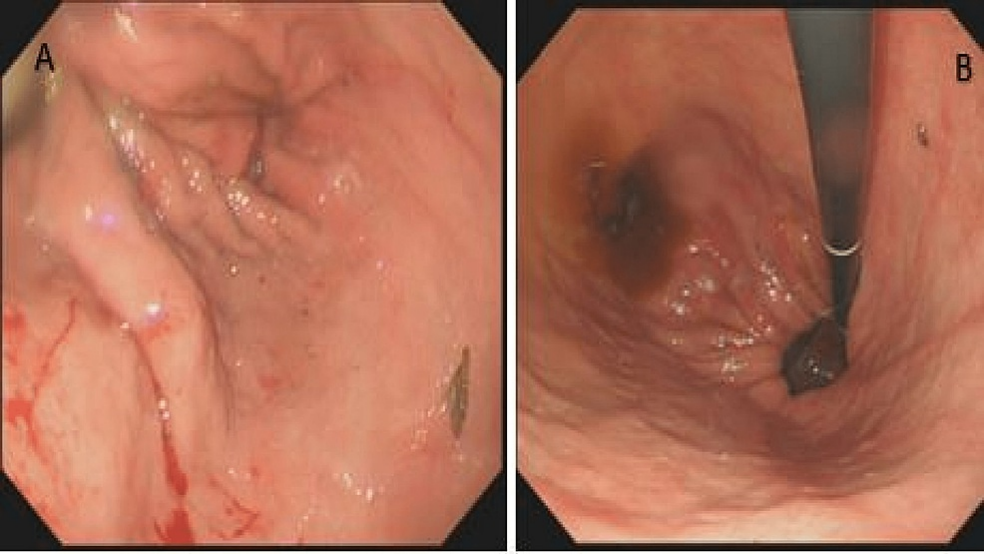
View of stomach following reduction of gastric volvulus via de-rotations. A) Antral view. B) Retroflexion view.

The gastric volvulus was finally reduced manually during endoscopy by de-rotation via continuous anticlockwise torquing of the endoscope (Figure 5).

Following, the patient was admitted to the gastroenterology ward for observation and ongoing intravenous saline infusion. The next day, she was started on a liquid diet followed by a soft diet. The possibility of recurrence of the condition was discussed with the patient and her family, and she declined any future surgical or laparoscopic management. She was discharged home after three days with the agreement for periodic gastroenterology clinic review. She was also started on oral omeprazole 20 milligrams once a day to be continued by her general practitioner.

## Discussion

Gastric volvulus is most commonly classified into organoaxial, mesenteroaxial, and combined [4]. Mesenteroaxial volvulus involves the horizontal axis along the greater and lesser stomach curvatures. This remains less common and is not associated with diaphragmatic defects [5]. Organoaxial rotation occurs along the longitudinal axis, namely the oesophagogastric junction and pylorus. It remains the most common form of gastric volvulus and found to be associated with diaphragmatic defects and its associated hiatus hernias [5]. It can have grave outcomes of incarceration and consequently increased mortality and morbidity [6].

The reported complications associated with acute gastric volvulus include aspiration, dehydration, gastric ischaemia with or without perforation, omental avulsion, pancreatitis, pancreatic necrosis [7] and splenic rupture from traction of splenic vessels [8]. Importantly, the abdominal examination may not reveal any guarding nor any peritonitis in cases where the entire stomach is sitting in the chest cavity [9] as in our patient. Chronic gastric volvulus presents with dysphagia, reflux symptoms that may be intermittent and chronic in nature, or there may be an acute or chronic presentation with a gastric outlet obstruction [10].

CT scans have been vital in providing a rapid diagnosis [11], although traditionally barium studies have equally been used to diagnose gastric volvulus [12] while a chest x-ray with air fluid level or upper abdominal bubble is helpful in initial investigations [12]. The management of gastric volvulus in the acute setting starts with intravenous fluid resuscitation, correction of electrolytes, and decompression via insertion of a nasogastric tube or Ryle’s tube [13].

Use of flexible endoscopy remains important as a diagnostic and therapeutic tool. It enables decompression of the stomach enabling the placement of a nasogastric tube, if previously not possible, and helps to restore the normal anatomic position via de-rotation. Lastly, it enables direct visualisation of the gastric mucosa to rule out any significant mucosal ischaemia [13]. Definitive treatments following identification of gastric ischaemia or to avoid future recurrence of gastric volvulus include laparoscopic hernial repair [14,15] and anterior abdominal wall gastropexy with placement of one or more percutaneous endoscopic gastrostomy (PEG) tubes [16,17]. However, for patients who are asymptomatic or with incidental findings of gastric volvulus the advocated approach is ‘watchful waiting’ [18]. This may be particularly relevant for patients with very high frailty scores or multiple comorbidities.

We attempted to highlight with this case the importance of going through a patient’s history of past medical illness. This, along with appropriate history taking followed by focussed examination, resulted in appropriate investigations being undertaken to confirm the diagnosis. This enabled the team to provide appropriate and timely management with a good outcome for this patient. It remains important to be more vigilant of gastric volvulus so timely intervention is possible.

## Conclusions

Different presentations with a variety of symptoms have been reported for gastric volvulus over the ages and it remains a diagnostic clinical challenge. This case report emphasises the importance of careful history taking, a review of past ailments and conditions and prompt clinical examination that enables the clinician to gauge rapid and appropriate investigations, orchestrate timely management and avoid potentially catastrophic outcomes. The understanding of a conservative approach in patients with significant comorbidities and reduced functional reserve remains vital. A prompt diagnosis would lead to timely management with better outcomes.
